# Public versus Private Drug Insurance and Outcomes of Patients Requiring Biologic Therapies for Inflammatory Bowel Disease

**DOI:** 10.1155/2017/7365937

**Published:** 2017-01-23

**Authors:** Amir Rumman, Roberto Candia, Justina J. Sam, Kenneth Croitoru, Mark S. Silverberg, A. Hillary Steinhart, Geoffrey C. Nguyen

**Affiliations:** ^1^Department of Medicine, University of Toronto, Toronto, ON, Canada; ^2^Departamento de Gastroenterología, Facultad de Medicina, Pontificia Universidad Católica de Chile, Santiago, Chile; ^3^Institute of Health Policy, Management and Evaluation, University of Toronto, Toronto, ON, Canada; ^4^Mount Sinai Hospital Centre for Inflammatory Bowel Disease, University of Toronto, Toronto, ON, Canada; ^5^Division of Gastroenterology, Department of Medicine, University of Toronto, Toronto, ON, Canada

## Abstract

*Background*. Antitumor necrosis factor (anti-TNF) therapy is a highly effective but costly treatment for inflammatory bowel disease (IBD).* Methods*. We conducted a retrospective cohort study of IBD patients who were prescribed anti-TNF therapy (2007–2014) in Ontario. We assessed if the insurance type was a predictor of timely access to anti-TNF therapy and nonroutine health utilization (emergency department visits and hospitalizations).* Results*. There were 268 patients with IBD who were prescribed anti-TNF therapy. Public drug coverage was associated with longer median wait times to first dose than private one (56 versus 35 days, *P* = 0.002). After adjusting for confounders, publicly insured patients were less likely to receive timely access to anti-TNF therapy compared with those privately insured (adjusted hazard ratio, 0.66; 95% CI: 0.45–0.95). After adjustment for demographic and clinical characteristics, publicly funded subjects were more than 2-fold more likely to require hospitalization (incidence rate ratio [IRR], 2.30; 95% CI: 1.19–4.43) and ED visits (IRR 2.42; 95% CI: 1.44–4.08) related to IBD.* Conclusions*. IBD patients in Ontario with public drug coverage experienced greater delays in access to anti-TNF therapy than privately insured patients and have a higher rate of hospitalizations and ED visits related to IBD.

## 1. Introduction

Crohn's disease (CD) and ulcerative colitis (UC) are two idiopathic inflammatory bowel diseases (IBD) characterized by chronic diarrhea, abdominal pain, and diminished quality of life [1, 2]. A third of patients with CD and 12% of those with UC require surgery within 5 years after diagnosis [3]. Currently, antitumor necrosis factor agents are among the most effective medical therapies for inducing and maintaining remission and achieving mucosal healing for CD and UC [1, 2, 4–7]. Though biologics have been conventionally indicated in individuals who are steroid-refractory or dependent and have failed immunomodulator therapy, there is growing evidence that biologic therapy may be more effective if given earlier in the course of disease [8, 9]. Moreover, biologic therapy has been associated with reduced hospitalizations and surgery [10–12].

The major barrier for utilization of biologic therapy is its high cost. In Ontario, the exceptional access program (EAP) is administered by the Ministry of Health and Long-term Care and facilities access to biologic therapies for IBD patients that lack supplementary private drug coverage. The EAP reimbursement criteria for initial approval of infliximab or adalimumab for Crohn's disease and ulcerative colitis patients are shown in Table 1. For patients with private drug insurance, coverage criteria for initial approval of infliximab or adalimumab vary significantly based on the insurance provider.

Although both systems enable access to biologic therapy, many gastroenterologists in Ontario feel that the type of coverage influences the wait time between prescribing the drug and its initiation. Furthermore, drug coverage status may be associated with differences in disease progression and health services utilization. The main aim of this study is to quantitatively evaluate if the type of drug insurance coverage is an independent predictor of the waiting time between prescription and administration of anti-TNF therapy and whether this impacted resource utilization driven by poorly controlled disease.

## 2. Methods

### 2.1. Patients and Design

A retrospective cohort was recruited from the Mount Sinai Hospital, a large IBD tertiary referral centre. All adult patients (≥18 years) with CD or UC who were prescribed anti-TNF therapy from January 1st 2007 to June 30th 2014 were eligible for this study. These individuals were identified by chart review of consecutive patients attending the IBD clinics. During the recruitment period the available anti-TNF agents were infliximab and adalimumab. The EAP coverage criteria for these agents did not change during the study period.

### 2.2. Data Collection

Demographic characteristics, smoking status, type of drug coverage (private or public), family history of IBD (1st degree), and postal code were collected. Postal code was linked to neighborhood income quintile using the postal code conversion file. Furthermore, we collected clinical characteristics to assess the type, behavior, and activity of IBD. These included Montreal classification [13], steroid-dependent or steroid-refractory IBD, age at diagnosis of IBD, duration of the disease up to prescription of biologic therapy, prior use of immunomodulators (azathioprine, 6-mercaptopurine, or methotrexate), and previous IBD-related surgery.

The following information was collected about anti-TNF therapy: setting of initiation (outpatient versus inhospital) and time interval between prescription and administration of first dose (days). Additionally, we ascertained whether the anti-TNF therapy was initially funded through a pharmaceutical compassionate drug use program, which provides patients drug free of charge while awaiting approval by a private or public insurer.

The primary predictor was type of insurance categorized as private insurance versus public insurance. Those with private drug coverage who applied for supplemental coverage through the publicly funded program were classified as having private insurance. An IBD patient was deemed steroid-dependent if they: (1) required two or more corticosteroid courses within a 12-month period; (2) experienced disease relapse when the corticosteroid dose was reduced below 15 mg of prednisone daily or within 6 weeks after stopping corticosteroids. IBD was deemed steroid-refractory when patients did not respond to high-dose oral prednisone (40–60 mg/day or equivalent) within 30 days or the intravenous equivalent dosing for 7 days [14, 15]. The disease phenotype of IBD was categorized as aggressive in patients with extensive UC (versus left-sided/distal UC); those with stricturing or penetrating CD (versus inflammatory phenotype); or those who had prior IBD surgery.

We collected the following outcome measures: (1) admission to our hospital with an IBD-related diagnosis after the decision to start biologic therapy and (2) an IBD-related visit to our emergency department (ED) that did not result in admission. Patients at our IBD centre are instructed to go to our ED or IBD-related urgent care whenever possible. Data were collected using a standardized data collection form.

### 2.3. Statistical Analysis

All analyses were performed with Stata version 14.0 (Statacorp LP, College Station, TX). Categorical data were compared using the chi-square statistic. Ordinal and continuous variables with skewed distribution were compared using Wilcoxon rank sum test.

Subjects who had inpatient initiation anti-TNF were excluded from all analyses pertaining to time from prescription to administration of anti-TNF therapy. These subjects were excluded because the streamlined process for attaining anti-TNF therapy in hospital is substantially more rapid than in the outpatient setting and not dependent on type of insurance coverage. Survival analyses were conducted in which the time-to-event outcome was the time interval from first application for anti-TNF therapy to administration of first dose of anti-TNF. Kaplan-Meier curves were constructed for each payer group and the log-rank test was used to compare unadjusted time-to-event curves.

Unadjusted rates of IBD-related hospitalizations and ED visits were determined by dividing the total number of IBD-related hospitalization and ER events by the total person-time from prescription of anti-TNF to the end of the study period. Hospitalization and ED rates were compared between those with private and public drug coverage with the chi-square statistics.

### 2.4. Multivariable Analysis

A multivariable Cox proportional hazard model was conducted to assess whether the type of insurance payer was an independent predictor of the time interval between prescription and administration of anti-TNF agents while adjusting for age, age at diagnosis, sex, neighborhood income quintile, exposure to immunomodulators, history of steroid-dependent or steroid-refractory IBD, aggressiveness of IBD phenotype, and need for supplemental public drug coverage. The robust variance estimator was used to account for clustering by physicians. Poisson regression was performed to assess whether type of insurance payer was associated with hospitalizations and ER visits while adjusting for the same confounders as the Cox regression model.

### 2.5. Ethics

The study was approved by the research ethics board of the Mount Sinai Hospital of Toronto.

## 3. Results

Two hundred sixty-eight IBD patients (CD, 62%; UC, 38%) were prescribed biologic therapy during the study period. All patients in our study cohort eventually received biologic therapy. One hundred ninety-one (71%) had private drug coverage, while 77 (29%) had public drug insurance coverage. Infliximab was used more frequently than adalimumab (91% versus 9%). Of those with private drug insurance, 30 (16%) required supplemental public funding through the provincial EAP to assist with copayments. The baseline characteristics of the study population are summarized in Table 2. Sixty-three (24%) patients received their first anti-TNF dose while being in hospital, and 40 (15%) patients received their first dose through a compassionate drug use program. Publicly funded individuals were more likely than their privately funded counterparts to be recipients of compassionate use (23% versus 12%, *P* = 0.014). They were also more likely to have been treated with an immunomodulator at the time of application for anti-TNF therapy (71% versus 53%, *P* = 0.007). There was no difference in neighborhood income quintiles between the two groups.

In addition to comparing demographic and clinical characteristics based on insurance status, we compared these characteristics between patients who received the first dose of biologic therapy within 20 days of prescription (25th percentile) and those who received the first dose more than 60 days after prescription (75th percentile). There were no significant differences in any of the demographic or clinical characteristics analysed.

### 3.1. Time to First Dose of Anti-TNF Therapy

**Figure 1:**
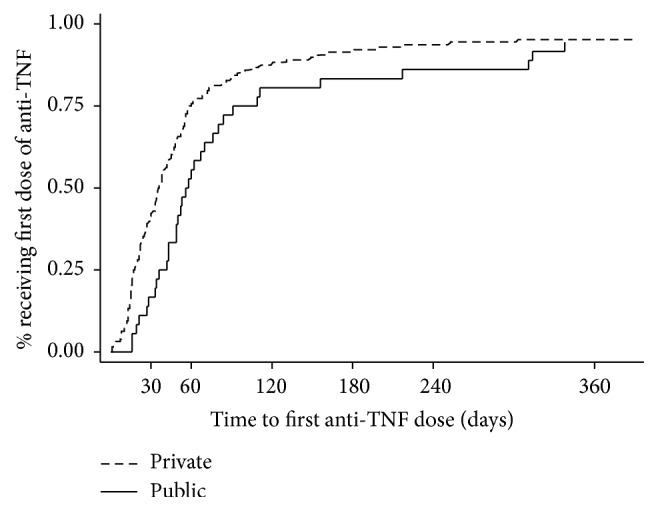
Kaplan-Meier curves for time to first dose of anti-TNF therapy stratified type of insurance drug coverage. Publicly funded subjects (solid line) experienced longer times to first anti-TNF dose than privately funded subjects (dashed line).

Figure 1 shows the Kaplan-Meier curves for receiving the first dose of anti-TNF therapy after exclusion of individuals who received their first dose in hospital. Patients with public drug insurance experienced greater delay in first dose administration compared to those with private insurance. The median time from prescription to first administration was 19 days longer for those with public drug insurance coverage (53 versus 34 days, *P* = 0.0216). After exclusion of patients enrolled in a compassionate drug use program, the median delay in starting anti-TNF therapy was 3 weeks longer in the public group (56 versus 35 days, *P* = 0.002).

The results of univariate and multivariable Cox regression analysis for the association between type of drug insurance coverage and time to first anti-TNF dose are shown in Table 3. After excluding patients who received their first anti-TNF dose while being in hospital, the adjusted hazard ratio (HR) for receiving the first dose of anti-TNF therapy in those with public versus private coverage was 0.66 (95% CI: 0.45–0.95, *P* = 0.026).

### 3.2. Hospitalizations and Emergency Department (ED) Visits

**Figure 2:**
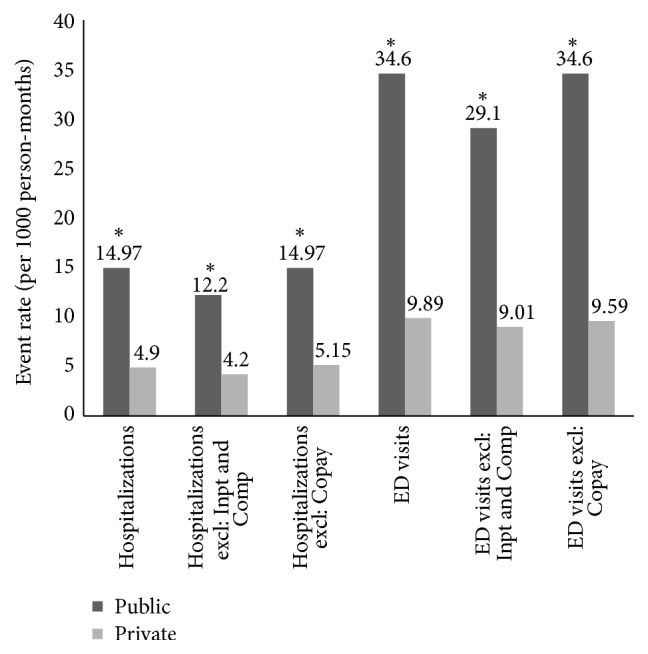
Rates of hospitalizations and emergency department visits stratified by public versus private drug insurance coverage. Additional sensitivity analyses are performed in which the following groups were excluded (excl): those who received first anti-TNF dose as inpatients (Inpt) or through a compassionate use program (Comp); those with private drug insurance coverage who received supplemental public funding (Copay). All rate differences between private and public drug coverage were statistically significant (^*∗*^*P* < 0.001).

A total of 126 IBD-related admissions and 272 ED visits occurred during the study period. Almost all IBD-related hospitalizations (120 of 126, 95%) and ED visits (268 of 272, 97%) occurred after the first dose of biologic therapy was given. Patients with public drug coverage experienced 3-fold higher rates of IBD-related hospitalizations than those privately insured (14.9 versus 4.91 hospitalizations per 1000 person-months, *P* < 0.001). Similarly, public drug coverage was associated with a 3-fold higher rate of IBD-related ED visits that did not result in hospitalization (34.6 versus 9.9 ED visits per 1000 person-months, *P* < 0.001) compared with private drug coverage. In sensitivity analyses, those who received anti-TNF in hospital, were recipients of compassionate drug use, or relied on supplemental public funding in addition to private drug insurance were excluded. With these exclusions, hospitalizations and ED visits remained 3-fold higher among those with public drug coverage compared with those private coverage (Figure 2).

After adjustment for age, gender, age at diagnosis, neighborhood income quintile, disease duration, disease subtype, history of immunomodulator therapy, history of steroid-refractory or steroid-dependent disease, and aggressive IBD phenotype, publicly funded subjects were more than 2-fold more likely to be hospitalized following the decision to start anti-TNF therapy (Table 4, incidence rate ratio [IRR], 2.30; 95% CI: 1.19–4.43, *P* = 0.013). Similarly, after controlling for the same confounders, those with public drug coverage were more than twice as likely to require an ED visit that did not lead to hospitalization (Table 5, IRR, 2.42; 95% CI: 1.44–4.08, *P* = 0.001). Additionally, female gender was an independent predictor of increased IBD-related hospitalization (IRR 2.96, 95% CI 1.60–5.47) and ED visits (IRR 1.83, 95% CI 1.03–3.27).

## 4. Discussion

Access to anti-TNF therapy is fundamental in the provision of high-quality care in IBD. In the Canadian province of Ontario, a government-funded and administered (public) program enables access to these costly medications when an IBD patient does not have sufficient private insurance coverage for prescription drug costs and is unable to pay out-of-pocket. Our study has demonstrated that delayed access to critical medical therapies may be driven by the type of drug insurance.

In our cohort, nearly a third of patients who require anti-TNF therapy required assistance through the provincial government's exceptional access program (EAP). In patients with private drug coverage, nearly a third required additional payment support in the form of compassionate access from the drug company or copay assistance. Furthermore, the lack of supplementary drug coverage was an independent predictor for increased utilization of nonroutine healthcare services. In our cohort, patients without private drug coverage were greater than 2-fold more likely to require IBD-related hospitalization and ED visits, after adjusting for demographic and clinical cofounders, including socioeconomic status. Our findings that most hospitalizations and ED visits occurred after the first dose of biologics was administered are worrisome. It suggests that perhaps there may be a durable impact of delayed access to biologics beyond drug initiation.

These findings are disconcerting in a system where all medically necessary health services are covered by a single payer but where prescription drug insurance coverage is not universally covered. In this system, reliance on public insurance may therefore introduce disparities in quality of care. Barriers to access may arise when the administrative review process frequently takes longer through the EAP program, often requiring multiple cycles of correspondence between the assessor and physician.

Physicians are able to partly compensate for delays in access to anti-TNF therapy through compassionate use programs funded by industry which essentially provide biologics free of charge for individuals who are unable to pay. Healthcare providers and patient advocacy groups should, therefore, lobby industry to sustain and expand compassionate use programs to reduce delays in access to biologics. Moreover, the recent introduction of a lower priced biosimilar of infliximab, which has been added to the Ontario Drug Formulary as a limited use (LU) drug product, will likely enhance access to anti-TNF therapy for those relying on public coverage. In Ontario, physicians are able to prescribe limited use drugs for specific clinical indications without having to navigate through the more complicated EAP review process.

As far as we are aware, to date there are no published studies assessing whether drug coverage is associated with delayed access to biologic therapy, adverse clinical outcomes, or increased utilization of healthcare studies. This holds true for IBD and other inflammatory conditions that require biologic therapy, such as rheumatoid arthritis, psoriasis, and psoriatic arthritis. This is the first study assessing these concerns in a universal healthcare system.

A major limitation of our study is that subjects were from a single tertiary centre, thus reducing our findings' generalizability. As a referral centre, we manage a higher proportion of complex IBD patients compared to most community practices and our patient population has high rate of biologic therapy use. The high volumes of patients on anti-TNF therapy at our institution reflect our experience in facilitating access to these drugs, especially for those requiring public coverage. Thus, insurance-based disparities in our study may underestimate the barriers in access experienced by community gastroenterology practices. Moreover, our findings reflect access patterns in Ontario but may not be generalizable to other provinces. Because Ontario accounts for more than a third of the Canadian population, issues surrounding medication access in this province certainly have national impact.

Furthermore, in assessing healthcare utilization, we only captured IBD-related admissions and ED visits at our institution. This likely leads to underestimation of rates of hospitalization and ED visits as patients may have received care at other centres in the Greater Toronto Area. While we attempted to adjust for aggressive IBD phenotype and other indicators of disease severity, there may have been residual confounding that partially explains differences in hospitalizations and ED visits between payer types. An additional limitation is that we did not collect data on whether continuation of biologic therapy in the follow-up period differed by payer type.

In conclusion, this study provides evidence that the type of drug coverage is an independent predictor of access to biologic therapy in IBD patients from Ontario. Cost analyses are needed to assess the economic impact of delays to anti-TNF therapy. Moreover, these insurance-based disparities likely extend beyond IBD to other conditions that require use of costly biologic therapies, including a myriad of rheumatologic conditions. As physicians and health policy makers, we must contemplate whether Canada can be truly considered to have a universal healthcare system when prompt access to critical medications relies on private drug insurance coverage.

## Figures and Tables

**Table 1:** Exceptional access program (EAP) reimbursement criteria for initial approval for use in Crohn's disease and ulcerative colitis patients. These criteria did not change during the study period.

Reimbursement criteria	Covered drugs
Treatment of fistulising Crohn's disease in patients with actively draining perianal disease or enterocutaneous fistula(e) that have recurred or persisted despite a course of antibiotic therapy (ciprofloxacin and/or metronidazole) and immunosuppressive therapy (azathioprine or 6-mercaptopurine).	Infliximab (Remicade®) Adalimumab (Humira®)

Treatment of moderate to severe (luminal) Crohn's disease in patients who have (i) Harvey-Bradshaw Index (HBI) score ≥ 7, (ii) failed to respond to conventional treatment with corticosteroids (prednisone 40 mg/day or equivalent for at least two weeks) or dose cannot be tapered below prednisone 20 mg/day or equivalent, (iii) failed to respond to an immunosuppressive agent (azathiopurine, 6-mercaptopurine, methotrexate, or cyclosporine) tried for at least 3 months.	Infliximab (Remicade®) Adalimumab (Humira®)

Mild ulcerative colitis: (i) Mayo score < 6; (ii) patients with mild disease will be considered on a case-by-case basis but submission must include rationale for coverage.	Infliximab (Remicade®)

Moderate ulcerative colitis: (i) Mayo score between 6 and 10 (inclusive), (ii) endoscopic subscore of 2, (iii) failed 2 weeks of oral prednisone 40 mg/day (or IV equivalent for at least 1 week) and 3 months of azathioprine or 6-mercaptopurine, or where the use of immunosuppressants is contraindicated, (iv) stabilized with 2 weeks of oral prednisone ≥ 40 mg/day (or IV equivalent for at least 1 week) but the prednisone dose cannot be tapered despite 3 months of azathioprine or 6-mercaptopurine, or where the use of immunosuppressants is contraindicated.	Infliximab (Remicade®)

Severe ulcerative colitis: (i) Mayo score between >10, (ii) endoscopic subscore of 2, (iii) failed 2 weeks of oral prednisone ≥ 40 mg/day (or IV equivalent for at least 1 week), (iv) stabilized with 2 weeks of oral prednisone ≥ 40 mg/day (or IV equivalent for at least 1 week) but the prednisone dose cannot be tapered despite 3 months of azathioprine or 6-mercaptopurine, or where the use of immunosuppressants is contraindicated.	Infliximab (Remicade®)

**Table 2:** Demographic and clinical characteristics.

Characteristic	All patients (*n* = 268)	Type of drug insurance coverage		*P* value
		Public (*n* = 77)	Private (*n* = 191)	
*Age at study inclusion*, years. Mean (SD)	32.5 (12.6)	31.8 (13.7)	32.8 (12.1)	0.548
*Age at IBD diagnosis*, years. Mean (SD)	24.2 (11.4)	25.4 (12.9)	23.7 (10.7)	0.316
*Disease duration*, years. Median (IQR)	8.5 (8.8)	6.9 (6.8)	9.1 (9.5)	0.247
*Male Gender*. *n* (%)	136 (51)	38 (49)	98 (51)	0.772
*Family history of IBD*. *n* (%)	47 (18)	13 (17)	34 (18)	0.858
*Smoking status*. *n* (%)				
Never	215 (80)	63 (82)	152 (80)	0.677
Former or Current	53 (20)	14 (18)	39 (20)	
*Neighborhood income quintile*. *n* (%)				0.880
(1)	29 (11)	9 (12)	20 (10)	
(2)	38 (14)	13 (17)	25 (13)	
(3)	57 (22)	16 (21)	41 (22)	
(4)	56 (21)	17 (22)	39 (22)	
(5)	83 (32)	21 (28)	62 (28)	
*Steroid dependent/refractory*. *n* (%)	148 (55)	49 (64)	99 (52)	0.079
*Previous/current immunomodulator*. *n* (%)				
Thiopurines	147 (55)	51 (66)	96 (50)	0.017
Methotrexate	28 (10)	9 (12)	19 (10)	0.673
Any immunomodulator	157 (59)	55 (71)	102 (53)	0.007
*IBD type*. *n* (%)				
Crohn's disease	165 (62)	48 (62)	117 (61)	0.869
Ulcerative colitis	103 (38)	29 (38)	74 (39)	
*Montreal classification, Crohn's disease*. *n* (%)				0.115
B1 (nonstricturing, nonpenetrating)	82 (50)	30 (62)	52 (45)	
B2 (stricturing)	52 (32)	12 (25)	40 (35)	
B3 (penetrating)	30 (18)	6 (13)	24 (21)	
*Extension of Ulcerative colitis*. *n* (%)				
Extensive or Pancolitis	78 (76)	22 (76)	56 (76)	0.989
Distal (left-sided/proctitis)	25 (24)	7 (24)	18 (24)	
*IBD of aggressive behavior*. *n* (%)	168 (63)	50 (65)	127 (67)	0.808
*Previous surgery*. *n* (%)	60 (22)	14 (18)	46 (24)	0.294
Crohn's disease	54 (33)	13 (27)	41 (35)	0.322
Ulcerative colitis	6 (6)	1 (3)	5 (7)	0.519
*Biologic agent initiated*. *n* (%)				
Infliximab	243 (91)	70 (91)	173 (91)	0.932
Adalimumab	25 (9)	7 (9)	18 (9)	
*Inpatient initiation of anti-TNF*. *n* (%)	63 (24)	24 (31)	39 (20)	0.079
*Compassionate use program*. *n* (%)	40 (15)	18 (23)	22 (12)	0.014
*Use of co-pay scheme*. *n* (%)	30 (11)	N/A	30 (16)	N/A

**Table 3:** Results of the Cox regression analysis of type of drug insurance coverage and time to first anti-TNF dose.

Characteristic	Hazard ratio	95% CI
Public drug coverage	0.67	0.45–0.95
Age	1.00	0.98–1.03
Age at IBD diagnosis	1.00	0.98–1.03
Female gender	1.10	0.78–1.56
Crohn's disease	1.46	1.00–2.12
Immunomodulator use	1.16	0.82–1.65
Steroid dependent or refractory disease	1.02	0.74–1.41
IBD of aggressive behaviour	0.81	0.55–1.18
Copay scheme use	0.74	0.37–1.48

**Table 4:** Poisson regression model for IBD-related admissions.

Characteristic	Unadjusted IRR (95% CI)	Adjusted IRR (95% CI)
Type of drug coverage		
Private	ref	ref
Public	2.32 (1.27–4.25)	2.30 (1.19–4.43)
Age	0.99 (0.97–1.02)	1.00 (0.97–1.03)
Age at IBD diagnosis	0.99 (0.97–1.02)	0.99 (0.96–1.03)
Sex		
Male	ref	ref
Female	2.97 (1.63–5.42)	2.96 (1.60–5.47)
IBD subtype		
Crohn's disease	ref	ref
Ulcerative colitis	0.77 (0.39–1.52)	0.75 (0.37–1.53)
Immunomodulator use	1.52 (0.79–2.94)	1.28 (0.67–2.46)
Steroid dependent or refractory	1.68 (0.92–3.06)	1.76 (1.01–3.05)
Aggressive IBD phenotype	0.86 (0.45–1.63)	1.15 (0.60–2.20)
Median neighborhood income		
1st quintile	ref	ref
2nd quintile	1.10 (0.43–2.81)	1.21 (0.53–2.75)
3rd quintile	0.62 (0.22–1.74)	0.77 (0.32–1.84)
4th quintile	0.35 (0.12–1.01)	0.46 (0.17–1.26)
5th quintile	0.83 (0.33–2.12)	0.96 (0.43–2.12)

**Table 5:** Poisson regression model for IBD-related ED visits.

Characteristic	Unadjusted IRR (95% CI)	Adjusted IRR (95% CI)
Type of drug coverage		
Private	ref	ref
Public	2.67 (1.57–4.55)	2.42 (1.43–4.08)
Age	0.99 (0.97–1.01)	0.99 (0.97–1.01)
Age at IBD diagnosis	0.99 (0.96–1.02)	0.99 (0.96–1.03)
Sex		
Male	ref	ref
Female	1.87 (1.07–3.26)	1.83 (1.03–3.27)
IBD subtype		
Crohn's disease	ref	ref
Ulcerative colitis	0.63 (0.32–1.24)	0.69 (0.35–1.36)
Immunomodulator use	2.30 (1.29–4.09)	1.87 (1.07–3.27)
Steroid dependent or refractory	1.59 (0.93–2.70)	1.48 (0.90–2.45)
Aggressive IBD phenotype	0.75 (0.43–1.30)	0.59 (0.20–1.80)
Median neighborhood income		
1st quintile	ref	ref
2nd quintile	0.75 (0.30–1.85)	0.76 (0.35–1.65)
3rd quintile	0.57 (0.24–1.35)	0.64 (0.30–1.38)
4th quintile	0.35 (0.12–1.00)	0.43 (0.17–1.13)
5th quintile	0.81 (0.35–1.90)	0.88 (0.40–1.94)
